# Authentic Leadership and Conflict‐Management Styles Among Nurse Leaders in Saudi Arabia: A Cross‐Sectional Study

**DOI:** 10.1155/jonm/7569784

**Published:** 2026-11-03

**Authors:** Bayan Alilyyani

**Affiliations:** ^1^ Department of Nursing Management and Education College of Nursing Taif University 2425 Taif Saudi Arabia tu.edu.sa

**Keywords:** authentic, conflict, leader, leadership, management, manager, nursing, Saudi Arabia, styles

## Abstract

**Background:**

Conflict is an inherent feature of nursing practice and may arise from competing demands, diverse professional roles, resource constraints, and pressures in clinical decision‐making. Nurse leaders play an important role in managing workplace conflict and selecting conflict‐management approaches appropriate to different situations. Authentic leadership, characterized by relational transparency, ethical decision‐making, balanced processing, and self‐awareness, may be relevant to how nurse leaders approach and manage workplace conflict.

**Aims:**

This study aimed to examine the relationship between authentic leadership and conflict‐management styles among nurse leaders in Saudi Arabia. Specifically, it examined the associations between authentic leadership and collaborating, accommodating, competing, avoiding, and compromising conflict‐management styles.

**Methods:**

A cross‐sectional survey was conducted among 96 nurse leaders working in public healthcare institutions in Saudi Arabia. Data were collected using a demographic questionnaire, the Rahim Organizational Conflict Inventory–II, and the Authentic Leadership Questionnaire. Data were analyzed using descriptive statistics, Pearson correlations, and linear regression, with multivariable adjustment for selected covariates and false discovery rate correction for exploratory analyses.

**Results:**

Overall conflict management was significantly and positively associated with authentic leadership (*β* = 0.469, *R* ^2^ = 0.220, *p* < 0.001). Significant positive associations were also observed for collaborating (*β* = 0.500, *R* ^2^ = 0.250, *p* < 0.001), accommodating (*β* = 0.431, *R* ^2^ = 0.186, *p* < 0.001), avoiding (*β* = 0.444, *R* ^2^ = 0.197, *p* < 0.001), and compromising (*β* = 0.472, *R* ^2^ = 0.223, *p* < 0.001). In contrast, competing was not significantly associated with authentic leadership (*β* = 0.185, *R* ^2^ = 0.034, *p* = 0.071).

**Conclusions:**

Authentic leadership was positively associated with collaborating, accommodating, avoiding, and compromising conflict‐management styles, but not with competing. These findings indicate associations rather than causation and warrant further longitudinal research.

## 1. Introduction

Conflict is an inherent feature of healthcare work and is particularly relevant to nursing because nurses work in complex, interdependent environments characterized by competing demands, diverse professional roles, resource constraints, and pressure in clinical decision‐making [1–3]. When inadequately managed, workplace conflict may disrupt professional relationships and the nursing work environment. Nurse leaders therefore have an important responsibility for recognizing and managing conflict and selecting appropriate strategies according to the circumstances in which conflict occurs [4, 5].

Conflict management is not a single behavioral response. Rahim’s framework identifies five styles—collaborating, accommodating, competing, avoiding, and compromising—that reflect different approaches to balancing one’s own concerns with those of others [6]. Collaborating emphasizes cooperation and the integration of different perspectives, whereas accommodating prioritizes the concerns of others. Competing emphasizes one’s own position, avoiding involves withdrawing from or minimizing conflict, and compromising seeks a mutually acceptable middle ground. Because the appropriateness of these approaches may vary according to the situation, effective nursing leadership requires the capacity to manage conflict flexibly rather than relying on a single style [2].

Authentic leadership may be particularly relevant to how nurse leaders approach workplace conflict. Authentic leadership comprises four interrelated dimensions: relational transparency, internalized moral perspective, balanced processing, and self‐awareness [7, 8]. These characteristics emphasize openness in relationships, ethical decision‐making, consideration of multiple perspectives, and awareness of one’s own strengths and limitations. In nursing, authentic leadership has been associated with favorable work environment and workforce outcomes [9–12]. Conceptually, its emphasis on transparency, balanced consideration of different viewpoints, and ethical relationships may also be relevant to how nurse leaders respond to interpersonal and organizational conflict.

Despite the importance of both constructs to nursing management, their relationship has received limited attention in nursing research. Previous literature has examined conflict‐management strategies among nursing professionals and the outcomes associated with authentic leadership, but comparatively little is known about how authentic leadership relates to the different conflict‐management styles used by nurse leaders. This gap is particularly relevant in Saudi Arabia, where understanding leadership behaviors associated with conflict management may inform leadership development and strategies for supporting healthy nursing work environments. Examining these relationships may therefore provide evidence to guide nurse managers in understanding how authentic leadership characteristics coexist with different approaches to managing workplace conflict.

Accordingly, this study aimed to examine the relationship between authentic leadership and conflict‐management styles among nurse leaders in Saudi Arabia. Specifically, it examined the relationships between authentic leadership and collaborating, accommodating, competing, avoiding, and compromising conflict‐management styles.

### 1.1. Research Hypotheses

The following hypotheses were proposed:

 H1a: Authentic leadership is significantly associated with the collaborating style.
 H1b: Authentic leadership is significantly associated with the accommodating style.
 H1c: Authentic leadership is significantly associated with the competing style.
 H1d: Authentic leadership is significantly associated with the avoiding style.
 H1e: Authentic leadership is significantly associated with the compromising style.

## 2. Materials and Methods

### 2.1. Study Design

A quantitative, cross‐sectional survey design was used to examine associations between conflict‐management styles and authentic leadership at a single point in time. The study was reported in accordance with the Strengthening the Reporting of Observational Studies in Epidemiology (STROBE) guidelines for cross‐sectional studies.

### 2.2. Study Setting

This online cross‐sectional study used nonprobability convenience sampling to recruit self‐identified nurse leaders working in public healthcare institutions in Saudi Arabia. Recruitment occurred through WhatsApp, LinkedIn, email, and professional networks, without a defined sampling frame or known invitation denominator. Participants self‐reported their eligibility; employment status, leadership role, and institutional affiliation were not independently verified. Although recruitment extended across the five geographical regions, the sample was not nationally representative. Recruitment was not restricted to predetermined healthcare institutions. To maintain anonymity, the names of participants’ employing institutions and other identifying organizational information were not collected.

### 2.3. Study Sample and Eligibility Criteria

A nonprobability convenience sampling approach was used to recruit nurse leaders working in public healthcare institutions across Saudi Arabia. The final sample comprised 96 nurse leaders. Because the original sample size estimation was not based on a fully specified effect‐size and statistical‐power framework, a sensitivity power analysis was conducted using G^∗^Power version 3.1.9.7 to determine the minimum detectable association supported by the achieved sample. For a two‐tailed Pearson correlation with *N* = 96, *α* = 0.05, and 80% power, the minimum detectable effect was approximately *r* = 0.28, representing a small‐to‐moderate association. Because this sensitivity analysis was based on a bivariate correlation rather than population‐proportion estimation or multiple‐predictor regression, an assumed population size and number of predictors were not required.

Eligible participants were nurse leaders currently working in public healthcare institutions in Saudi Arabia, with at least 1 year of nursing leadership experience, who voluntarily agreed to participate and were able to complete the online questionnaire. Nurse leaders working in private healthcare institutions, those with less than 1 year of leadership experience, staff nurses and nursing assistants without leadership roles, nurse leaders who were on leave during the data‐collection period, and individuals unable to complete the online questionnaire were excluded.

Before accessing the study questionnaires, potential participants were presented with the eligibility criteria and asked to confirm that they were currently working as nurse leaders in a public healthcare institution in Saudi Arabia and had at least 1 year of nursing leadership experience. Eligibility was therefore determined by participant self‐report. Because the survey was anonymous and no identifying employment or institutional information was collected, participants’ employment status and institutional affiliation were not independently verified.

### 2.4. Instruments

Three self‐report instruments were used for data collection: a demographic questionnaire, the Rahim Organizational Conflict Inventory–II (ROCI‐II), and the Authentic Leadership Questionnaire (ALQ). All study questionnaires were administered in English; therefore, no translation or cultural‐adaptation procedures were undertaken for the present study.

#### 2.4.1. Demographic Questionnaire

Participant characteristics were collected using a researcher‐developed demographic questionnaire that included age, gender, nationality, highest level of nursing education, years of experience in nursing leadership, and geographical region of Saudi Arabia.

#### 2.4.2. Conflict Management

Conflict‐management styles were measured using the ROCI‐II [13]. The instrument consists of 28 items rated on a five‐point Likert scale from 1 (strongly disagree) to 5 (strongly agree) and comprises five subscales: collaborating (7 items), accommodating (6 items), competing (5 items), avoiding (6 items), and compromising (4 items). Scores for each conflict‐management style were calculated as the mean of the items comprising the respective subscale, with higher scores indicating greater use of that conflict‐management style. An overall conflict‐management score was calculated as the mean across all 28 items. Scores were treated as continuous variables, and no low, moderate, or high classifications were applied because predefined validated cutoff values were not used in this study. Higher scores indicate greater use of the respective conflict‐management style. Previous studies have supported the psychometric properties of the ROCI‐II. In the present sample, internal consistency was excellent for the total scale (Cronbach’s *α* = 0.965), collaborating (*α* = 0.920), and accommodating (*α* = 0.911), and good for competing (*α* = 0.828), avoiding (*α* = 0.890), and compromising (*α* = 0.891). No separate construct‐validity analysis was undertaken in the present study.

#### 2.4.3. Authentic Leadership

Authentic leadership was measured using the ALQ [14]. The instrument consists of 16 items across four dimensions: relational transparency (5 items), internalized moral perspective (4 items), balanced processing (3 items), and self‐awareness (4 items). Items are rated on a five‐point scale ranging from 0 (not at all) to 4 (frequently, if not always). Dimension scores were calculated as the mean of the items comprising each ALQ dimension, and the total authentic leadership score was calculated as the mean across all 16 items. Higher scores indicated greater levels of authentic leadership. Scores were treated as continuous variables, and no low, moderate, or high classifications were applied because predefined validated cutoff values were not used in this study. Permission to use the ALQ was obtained under the appropriate license (order number: 83,290). Previous studies have supported the psychometric properties of the instrument. In the present sample, internal consistency was excellent for the total scale (Cronbach’s *α* = 0.966) and self‐awareness (*α* = 0.941), and good for relational transparency (*α* = 0.880), internalized moral perspective (*α* = 0.888), and balanced processing (*α* = 0.800). No separate construct‐validity analysis was undertaken in the present study.

Both questionnaires were administered in English. Internal consistency was assessed in the present sample; however, no population‐specific construct‐validity or cross‐cultural measurement‐equivalence assessment was conducted. The reported Cronbach’s alpha coefficients therefore provide evidence of internal consistency rather than comprehensive validation for this multinational sample.

## 3. Data Collection Procedure

Data were collected from October 2025 to May 2026, following ethical approval. Participants were recruited through professional networks and electronic platforms, including institutional/professional email channels, WhatsApp groups, and LinkedIn. The first page of the online questionnaire provided information about the study, eligibility criteria, voluntary participation, confidentiality, and informed consent. Potential participants were required to confirm their eligibility and consent before proceeding to the study questionnaires. The total number of individuals who received or viewed the invitation was unknown because recruitment occurred through professional networks and electronic platforms without a defined invitation denominator; therefore, a response rate could not be calculated. A total of 100 individuals accessed the online questionnaire. Four submitted incomplete questionnaires and were excluded, leaving 96 complete questionnaires for analysis. The survey platform restricted repeat submissions to minimize duplicate responses. No identifying institutional information was collected to preserve participant anonymity.

### 3.1. Data Analysis

Data were analyzed using IBM SPSS Statistics version 28.0.1.1. Before analysis, the dataset was screened for completeness and data‐entry accuracy. Of the 100 individuals who accessed the online questionnaire, four submitted incomplete questionnaires and were excluded, resulting in a complete‐case analytical sample of 96 participants. Because the included cases had complete data for the study variables, no missing‐data imputation was performed.

Descriptive statistics were used to summarize participant characteristics and study variables. Categorical variables were reported as frequencies and percentages, whereas continuous variables were summarized using means and standard deviations. Internal consistency of the study measures was assessed using Cronbach’s alpha.

Pearson correlation analysis was used to examine the strength and direction of bivariate associations between authentic leadership and conflict‐management scores. Before inferential analysis, relevant statistical assumptions were evaluated. Linearity was assessed by visual inspection of scatterplots. For the regression models, normality of residuals was evaluated using normal probability plots and the distribution of standardized residuals, and homoscedasticity was assessed by examining plots of standardized residuals against standardized predicted values. Standardized residuals and Cook’s distance were examined to identify potentially influential observations. No other substantial assumption violations or influential observations requiring exclusion were identified.

Simple linear regression analyses were used to estimate the associations between conflict‐management scores and authentic leadership. In both simple and multivariable regression analyses, authentic leadership scores were specified as the dependent variables and conflict‐management scores as the predictors. The adjusted models used total authentic leadership as the dependent variable, with each conflict‐management style examined separately. This model specification was used to estimate associations and does not imply that conflict‐management styles causally influence authentic leadership. The associations between the five conflict‐management styles and overall authentic leadership were considered the principal hypothesis‐driven analyses. Additional analyses involving the four authentic leadership dimensions were considered exploratory. To account for multiple testing across the 20 exploratory dimension‐level analyses, the Benjamini–Hochberg false discovery rate (FDR) procedure was applied. Statistical significance for these exploratory analyses was evaluated using an FDR‐adjusted q value < 0.05. Both unadjusted *p* values and FDR‐adjusted q values were reported. Pearson correlation results were reported using the correlation coefficient (r), whereas regression results were reported using the unstandardized regression coefficient (B) with 95% confidence interval (CI), standardized regression coefficient (β), coefficient of determination (*R* ^2^), and *p* value. All statistical tests were two‐sided. Statistical significance for the principal hypothesis‐driven analyses was set at *p* < 0.05.

Age, gender, nationality, educational level, leadership experience, and geographical region were included as adjustment covariates because they could plausibly relate to both authentic leadership and conflict‐management styles. Age and leadership experience may reflect differences in professional maturity and exposure to leadership and conflict situations. Educational level may reflect differences in preparation for leadership, ethical decision‐making, and conflict management. Gender may capture differences in workplace expectations and professional experiences relevant to both constructs. Nationality may serve as an imperfect proxy for differences in cultural and professional socialization, while geographical region may capture variation in organizational and workforce contexts. These variables were treated as potential confounding factors rather than empirically established confounders, and adjustment was not assumed to eliminate residual confounding.

To examine whether the associations persisted after adjustment for selected demographic and professional characteristics, additional multivariable linear regression analyses were conducted. Each conflict‐management style was examined in a separate model adjusted for age, gender, nationality, educational level, leadership experience, and geographical region. To maintain model parsimony relative to the sample size, age, educational level, and leadership experience were entered as ordinal variables; gender was entered as a binary variable; nationality was categorized as Saudi versus non‐Saudi; and geographical region was categorized as Western versus non‐Western. Because diagnostic evaluation indicated some departure from homoscedasticity in selected models, HC3 heteroscedasticity‐robust standard errors were used for confidence intervals and statistical significance testing in the adjusted analyses. Adjusted unstandardized coefficients (B) with robust 95% confidence intervals, standardized coefficients (β), *R* ^2^, adjusted *R* ^2^, and two‐sided *p* values were reported.

### 3.2. Ethical Considerations

Ethical approval was obtained from the Scientific Research Ethics Committee at Taif University (IRB No. 46‐284; April 17, 2025). The study was conducted in accordance with the Declaration of Helsinki. Before completing the questionnaires, participants received information about the study background and objectives, eligibility criteria, their right to decline participation or withdraw without consequences, and the researcher’s contact details. Electronic informed consent was obtained before participants proceeded to the questionnaires. Data were collected anonymously; institutional names, affiliations, and participant contact information were not collected. The data were stored securely and were accessible only to the researcher.

## 4. Results

The analytical sample comprised 96 nurse leaders. The largest age group was 36–41 years (*n* = 33, 34.4%), and the smallest was 24–29 years (*n* = 11, 11.5%). Most participants were female (*n* = 59, 61.5%), and 52 (54.2%) were Saudi nationals. A bachelor’s degree in nursing was the most common qualification (*n* = 59, 61.5%); two participants (2.1%) held a PhD in nursing. More than 10 years of leadership experience was reported by 34 participants (35.4%), while the 4–6‐year and 7–9‐year groups each included 23 participants (24.0%). Most participants were from the Western region (*n* = 58, 60.4%), whereas six (6.3%) were from the Northern region. Participant characteristics are presented in Table 1.

**TABLE 1:** Participant characteristics (*N* = 96).

Demographics		Number	%
Age	Total	96	100
	24–29 years	11	11.5
	30–35 years	25	26.0
	36–41 years	33	34.4
	Over 41 years	27	28.1

Gender	Total	96	100
	Female	59	61.5
	Male	37	38.5

Nationality	Total	96	100
	Saudi	52	54.2
	Non‐Saudi	44	45.8

Highest level of nursing education	Total	96	100
	Bachelor’s degree in nursing	59	61.5
	Master’s degree in nursing	29	30.2
	Diploma in nursing	6	6.3
	PhD in nursing	2	2.1

Leadership experience	Total	96	100
	1–3 years	16	16.7
	4–6 years	23	24.0
	7–9 years	23	24.0
	10 years and more	34	35.4

Region	Total	96	100
	Central region	11	11.5
	Eastern region	13	13.5
	Northern region	6	6.3
	Southern region	8	8.3
	Western region	58	60.4

Descriptive statistics for the study variables are presented in Table 2. The mean total conflict‐management score was 3.72 (SD = 0.749). Among the five conflict‐management styles, collaborating had the highest mean score (*M* = 3.98, SD = 0.784), whereas competing had the lowest (*M* = 3.26, SD = 0.940). The mean total authentic leadership score was 3.21 (SD = 0.741), with mean scores across its four dimensions ranging from 3.17 (SD = 0.829) for internalized moral perspective to 3.27 (SD = 0.803) for self‐awareness. All study measures demonstrated good to excellent internal consistency (Cronbach’s *α* = 0.800–0.966; Table 2).

**TABLE 2:** Descriptive statistics and internal consistency of study variables.

Study variable	Mean	Standard deviation	Cronbach’s alpha
Total conflict‐management score	3.72	0.749	0.965
Collaborating style	3.98	0.784	0.920
Accommodating style	3.78	0.815	0.911
Competing style	3.26	0.940	0.828
Avoiding style	3.69	0.857	0.890
Compromising style	3.77	0.893	0.891
Total authentic leadership score	3.21	0.741	0.966
Relational transparency	3.19	0.764	0.880
Internalized moral perspective	3.17	0.829	0.888
Balanced processing	3.22	0.759	0.800
Self‐awareness	3.27	0.803	0.941

Pearson correlation analyses showed that total conflict management was positively correlated with total authentic leadership (*r* = 0.46, *p* < 0.001). Authentic leadership was also positively correlated with collaborating (*r* = 0.50, *p* < 0.001), accommodating (*r* = 0.43, *p* < 0.001), avoiding (*r* = 0.44, *p* < 0.001), and compromising (*r* = 0.47, *p* < 0.001). The correlation between competing and authentic leadership was weak and not statistically significant (*r* = 0.18, *p* > 0.05) (Table 3). Simple linear regression analyses showed that total conflict management was significantly associated with authentic leadership (*B* = 0.465, 95% CI [0.286, 0.644], *β* = 0.469, *R* ^2^ = 0.220, *p* < 0.001). Among the individual conflict‐management styles, collaborating showed the strongest association with authentic leadership (*B* = 0.472, 95% CI [0.304, 0.640], *β* = 0.500, *R* ^2^ = 0.250, *p* < 0.001). Accommodating (*B* = 0.392, 95% CI [0.224, 0.560], *β* = 0.431, *R* ^2^ = 0.186, *p* < 0.001), avoiding (*B* = 0.384, 95% CI [0.225, 0.543], *β* = 0.444, *R* ^2^ = 0.197, *p* < 0.001), and compromising (*B* = 0.392, 95% CI [0.242, 0.542], *β* = 0.472, *R* ^2^ = 0.223, *p* < 0.001) were also significantly associated with authentic leadership. Competing was not significantly associated with authentic leadership (*B* = 0.146, 95% CI [−0.013, 0.305], *β* = 0.185, *R* ^2^ = 0.034, *p* = 0.071) (Table 4).

**TABLE 3:** Correlations among study variables.

Study variable	CMS	CS1	AS1	CS2	AS2	CS3	AL	T	M/E	BP	SA
CMS	—										
CS1	0.86^∗∗∗^	—									
AS1	0.94^∗∗∗^	0.77^∗∗∗^	—								
CS2	0.73^∗∗∗^	0.46^∗∗∗^	0.64^∗∗∗^	—							
AS2	0.93^∗∗∗^	0.76^∗∗∗^	0.90^∗∗∗^	0.58^∗∗∗^	—						
CS3	0.91^∗∗∗^	0.78^∗∗∗^	0.84^∗∗∗^	0.59^∗∗∗^	0.87^∗∗∗^	—					
AL	0.46^∗∗∗^	0.50^∗∗∗^	0.43^∗∗∗^	0.18	0.44^∗∗∗^	0.47^∗∗∗^	—				
T	0.43^∗∗∗^	0.46^∗∗∗^	0.40^∗∗∗^	0.17	0.43^∗∗∗^	0.41^∗∗∗^	0.93^∗∗∗^	—			
M/E	0.36^∗∗∗^	0.40^∗∗∗^	0.32^∗∗^	0.13	0.35^∗∗∗^	0.38^∗∗∗^	0.94^∗∗∗^	0.85^∗∗∗^	—		
BP	0.47^∗∗∗^	0.51^∗∗∗^	0.40^∗∗∗^	0.23^∗^	0.41^∗∗∗^	0.49^∗∗∗^	0.92^∗∗∗^	0.79^∗∗∗^	0.84^∗∗∗^	—	
SA	0.48^∗∗∗^	0.51^∗∗∗^	0.47^∗∗∗^	0.17	0.45^∗∗∗^	0.49^∗∗∗^	0.92^∗∗∗^	0.80^∗∗∗^	0.80^∗∗∗^	0.88^∗∗∗^	—

*Note:* CS1, collaborating style; AS1, accommodating style; CS2, competing style; AS2, avoiding style; CS3, compromising style; T, transparency; M/E; moral/ethical.

Abbreviations: AL, authentic leadership; BP, balanced processing; CMS, conflict‐management styles; SA, self‐awareness.

^∗^ *p* < 0.05.

^∗∗^ *p* < 0.01.

^∗∗∗^ *p* < 0.001.

**TABLE 4:** Simple linear regression associations between conflict management and authentic leadership.

Predictor ⟶ outcome	*R* ^2^	B	β	95% confidence interval		*p*	FDR‐adjusted *q*
				Lower	Upper		
CMS ⟶ AL	0.220	0.465	0.469	0.286	0.644	< 0.001	—
CMS ⟶ T	0.191	0.446	0.437	0.258	0.635	< 0.001	—
CMS ⟶ M/E	0.136	0.417	0.369	0.202	0.632	< 0.001	—
CMS ⟶ BP	0.223	0.479	0.472	0.296	0.662	< 0.001	—
CMS ⟶ SA	0.239	0.525	0.489	0.333	0.717	< 0.001	—
CS1 ⟶ AL	0.250	0.472	0.500	0.304	0.640	< 0.001	—
CS1 ⟶ T	0.212	0.449	0.461	0.272	0.626	< 0.001	< 0.001
CS1 ⟶ M/E	0.161	0.432	0.401	0.230	0.634	< 0.001	< 0.001
CS1 ⟶ BP	0.265	0.498	0.515	0.328	0.668	< 0.001	< 0.001
CS1 ⟶ SA	0.260	0.522	0.510	0.341	0.702	< 0.001	< 0.001
AS1 ⟶ AL	0.186	0.392	0.431	0.224	0.560	< 0.001	—
AS1 ⟶ T	0.166	0.382	0.408	0.207	0.558	< 0.001	< 0.001
AS1 ⟶ M/E	0.106	0.337	0.325	0.137	0.538	0.001	0.002
AS1 ⟶ BP	0.165	0.378	0.406	0.204	0.552	< 0.001	< 0.001
AS1 ⟶ SA	0.228	0.470	0.477	0.293	0.647	< 0.001	< 0.001
CS2 ⟶ AL	0.034	0.146	0.185	−0.013	0.305	0.071	—
CS2 ⟶ T	0.030	0.141	0.173	−0.023	0.305	0.091	0.096
CS2 ⟶ M/E	0.017	0.117	0.131	−0.065	0.300	0.205	0.205
CS2 ⟶ BP	0.053	0.186	0.230	0.025	0.346	0.024	0.028
CS2 ⟶ SA	0.031	0.151	0.177	−0.021	0.323	0.085	0.094
AS2 ⟶ AL	0.197	0.384	0.444	0.225	0.543	< 0.001	—
AS2 ⟶ T	0.186	0.385	0.432	0.220	0.550	< 0.001	< 0.001
AS2 ⟶ M/E	0.125	0.349	0.354	0.160	0.538	< 0.001	< 0.001
AS2 ⟶ BP	0.172	0.367	0.415	0.202	0.532	< 0.001	< 0.001
AS2 ⟶ SA	0.210	0.430	0.458	0.259	0.600	< 0.001	< 0.001
CS3 ⟶ AL	0.223	0.392	0.472	0.242	0.542	< 0.001	—
CS3 ⟶ T	0.170	0.354	0.413	0.194	0.513	< 0.001	< 0.001
CS3 ⟶ M/E	0.149	0.366	0.387	0.187	0.545	< 0.001	< 0.001
CS3 ⟶ BP	0.243	0.419	0.493	0.268	0.571	< 0.001	< 0.001
CS3 ⟶ SA	0.245	0.445	0.495	0.285	0.605	< 0.001	< 0.001

*Note:* CMS = total conflict‐management score; CS1 = collaborating; AS1 = accommodating; CS2 = competing; AS2 = avoiding; CS3 = compromising; T = relational transparency; M/E = internalized moral perspective; B = unstandardized regression coefficient; β = standardized regression coefficient. The five individual conflict‐management style associations with overall authentic leadership were considered the principal hypothesis‐driven analyses. Analyses involving the five conflict‐management styles and four authentic leadership dimensions were considered exploratory; *p* values for these 20 comparisons were adjusted using the Benjamini–Hochberg FDR procedure. Statistical significance was evaluated at *p* < .05 for the principal analyses and *q* < .05 for the exploratory analyses. Dashes indicate analyses not included in the FDR‐adjusted exploratory family.

Abbreviations: AL, authentic leadership; BP, balanced processing; CI, confidence interval; FDR, false discovery rate; SA, self‐awareness.

In the exploratory dimension‐level analyses, relational transparency was positively associated with collaborating (*B* = 0.449, 95% CI [0.272, 0.626], *β* = 0.461, *R* ^2^ = 0.212), accommodating (*B* = 0.382, 95% CI [0.207, 0.558], *β* = 0.408, *R* ^2^ = 0.166), avoiding (*B* = 0.385, 95% CI [0.220, 0.550], *β* = 0.432, *R* ^2^ = 0.186), and compromising (*B* = 0.354, 95% CI [0.194, 0.513], *β* = 0.413, *R* ^2^ = 0.170), with all associations remaining significant after FDR adjustment (*q* < 0.001). Competing was not significantly associated with relational transparency (*B* = 0.141, 95% CI [−0.023, 0.305], *β* = 0.173, *R* ^2^ = 0.030, *p* = 0.091, *q* = 0.096).

Internalized moral perspective was positively associated with collaborating (*B* = 0.432, 95% CI [0.230, 0.634], *β* = 0.401, *R* ^2^ = 0.161, *q* < 0.001), accommodating (*B* = 0.337, 95% CI [0.137, 0.538], *β* = 0.325, *R* ^2^ = 0.106, *p* = 0.001, *q* = 0.002), avoiding (*B* = 0.349, 95% CI [0.160, 0.538], *β* = 0.354, *R* ^2^ = 0.125, *q* < 0.001), and compromising (*B* = 0.366, 95% CI [0.187, 0.545], *β* = 0.387, *R* ^2^ = 0.149, *q* < 0.001). Competing was not significantly associated with internalized moral perspective (*B* = 0.117, 95% CI [−0.065, 0.300], *β* = 0.131, *R* ^2^ = 0.017, *p* = 0.205, *q* = 0.205).

Balanced processing was positively associated with collaborating (*B* = 0.498, 95% CI [0.328, 0.668], *β* = 0.515, *R* ^2^ = 0.265), accommodating (*B* = 0.378, 95% CI [0.204, 0.552], *β* = 0.406, *R* ^2^ = 0.165), avoiding (*B* = 0.367, 95% CI [0.202, 0.532], *β* = 0.415, *R* ^2^ = 0.172), and compromising (*B* = 0.419, 95% CI [0.268, 0.571], *β* = 0.493, *R* ^2^ = 0.243), with all associations remaining significant after FDR adjustment (*q* < 0.001). Competing also showed a modest positive association with balanced processing (*B* = 0.186, 95% CI [0.025, 0.346], *β* = 0.230, *R* ^2^ = 0.053, *p* = 0.024, FDR‐adjusted *q* = 0.028).

Finally, self‐awareness was positively associated with collaborating (*B* = 0.522, 95% CI [0.341, 0.702], *β* = 0.510, *R* ^2^ = 0.260), accommodating (*B* = 0.470, 95% CI [0.293, 0.647], *β* = 0.477, *R* ^2^ = 0.228), avoiding (*B* = 0.430, 95% CI [0.259, 0.600], *β* = 0.458, *R* ^2^ = 0.210), and compromising (*B* = 0.445, 95% CI [0.285, 0.605], *β* = 0.495, *R* ^2^ = 0.245), with all associations remaining significant after FDR adjustment (*q* < 0.001). Competing was not significantly associated with self‐awareness (*B* = 0.151, 95% CI [−0.021, 0.323], *β* = 0.177, *R* ^2^ = 0.031, *p* = 0.085, *q* = 0.094).

Multivariable linear regression analyses using HC3 heteroscedasticity‐robust standard errors were conducted to examine whether the principal associations persisted after adjustment for age, gender, nationality, educational level, leadership experience, and geographical region (Table 5). Collaborating remained positively associated with authentic leadership (*B* = 0.493, robust 95% CI [0.266, 0.720], *β* = 0.521, *p* < 0.001), as did accommodating (*B* = 0.386, robust 95% CI [0.172, 0.600], *β* = 0.425, *p* < 0.001), avoiding (*B* = 0.383, robust 95% CI [0.181, 0.585], *β* = 0.443, *p* < 0.001), and compromising (*B* = 0.383, robust 95% CI [0.154, 0.612], *β* = 0.461, *p* = 0.001). Competing was not significantly associated with authentic leadership after covariate adjustment (*B* = 0.160, robust 95% CI [−0.050, 0.369], *β* = 0.203, *p* = 0.133). Thus, adjustment for the selected demographic and professional characteristics did not materially alter the overall pattern of the principal findings.

**TABLE 5:** Multivariable associations between conflict‐management styles and authentic leadership using HC3 robust standard errors.

Conflict‐management style	Adjusted *B*	Robust 95% CI	Adjusted β	*R* ^2^	Adjusted *R* ^2^	Robust *p*
Collaborating	0.493	[0.266, 0.720]	0.521	0.267	0.209	< 0.001
Accommodating	0.386	[0.172, 0.600]	0.425	0.208	0.145	< 0.001
Competing	0.160	[−0.050, 0.369]	0.203	0.073	−0.000	0.133
Avoiding	0.383	[0.181, 0.585]	0.443	0.224	0.163	< 0.001
Compromising	0.383	[0.154, 0.612]	0.461	0.240	0.180	0.001

*Note: B* = unstandardized regression coefficient; β = standardized regression coefficient. Authentic leadership was the dependent variable. Each conflict‐management style was examined in a separate multivariable linear regression model adjusted for age, gender, nationality, educational level, leadership experience, and geographical region. Age, educational level, and leadership experience were entered as ordinal variables; gender was entered as a binary variable; nationality was categorized as Saudi versus non‐Saudi; and geographical region was categorized as Western versus non‐Western. HC3 heteroscedasticity‐robust standard errors were used for confidence intervals and hypothesis tests. *R* ^2^ and adjusted *R* ^2^ refer to the corresponding ordinary least‐squares model.

Abbreviation: CI, confidence interval.

## 5. Discussion

This study examined the relationships between authentic leadership and conflict‐management styles among nurse leaders in Saudi Arabia. Authentic leadership was positively associated with overall conflict management and with collaborating, accommodating, avoiding, and compromising styles, whereas its association with competing was not statistically significant. The positive associations for collaborating, accommodating, avoiding, and compromising remained statistically significant after adjustment for selected demographic and professional characteristics. Collectively, these findings suggest that authentic leadership characteristics coexist with multiple approaches to conflict management rather than corresponding to a single conflict‐management style.

The observed pattern can be interpreted through the four components of authentic leadership: self‐awareness, relational transparency, internalized moral perspective, and balanced processing [7, 8]. These components provide a theoretical basis for understanding how authentic leadership may relate to different approaches to conflict. Self‐awareness involves recognition of one’s strengths, limitations, values, and responses; relational transparency emphasizes openness and authenticity in interpersonal relationships; internalized moral perspective reflects behavior guided by internal ethical standards; and balanced processing involves objectively considering relevant information and alternative perspectives before making decisions. Importantly, these characteristics do not prescribe a single behavioral response to every conflict. Rather, they may shape how leaders understand, evaluate, and respond to conflict while allowing different strategies to be used according to situational demands. Thus, the associations observed in this study are theoretically compatible with a context‐sensitive view of conflict management, although the cross‐sectional design does not establish that authentic leadership determines the conflict‐management style selected by nurse leaders.

Among the conflict‐management styles, collaborating had the highest mean score, followed by accommodating, compromising, avoiding, and competing. This pattern is partly consistent with previous nursing literature. Labrague et al. [2] identified collaborating and accommodating as commonly used approaches to conflict management among nursing professionals, whereas avoiding and competing were less frequently used. Similarly, Sharma et al. [15] reported accommodating, compromising, and collaborating as the commonly used styles among newly employed nurses, with competing and avoiding used less frequently. Findings across settings, however, are not uniform. Delak and Širok [16] reported avoidance as a prominent conflict‐resolution approach among nurses. These variations suggest that conflict‐management behavior may differ according to professional role, organizational environment, interpersonal relationships, and the circumstances surrounding the conflict rather than reflecting a universally preferred pattern.

The mean authentic leadership score in the present study was 3.21 (SD = 0.741), with relatively similar scores across relational transparency, internalized moral perspective, balanced processing, and self‐awareness. Because validated cutoff values were not applied, these scores should not be categorized as indicating high, moderate, or low authentic leadership. Nevertheless, the observed mean can be considered in relation to previous studies. Metwally et al. [17] reported a mean authentic leadership score of 3.38 (SD = 1.23) in an Egyptian nursing context, whereas Allan and Rayan [10] reported a mean of 2.08 (SD = 0.87) among participants in Jordan. Differences between studies should be interpreted cautiously because they may reflect variations in participant characteristics, healthcare systems, leadership roles, organizational environments, and study procedures.

Authentic leadership was positively associated with the overall conflict‐management score. This finding is broadly consistent with previous evidence indicating relationships between authentic leadership characteristics and approaches to conflict management. Villarreal [18] reported significant relationships involving relational transparency, internalized moral perspective, self‐awareness, and conflict management, while Demirdag and Kalafat [19] identified relationships between authentic leadership and conflict‐management styles in an educational setting. Theoretically, the present association may reflect the relevance of self‐awareness, transparent interpersonal relationships, ethical decision‐making, and balanced consideration of information when leaders encounter disagreement. However, these findings should not be interpreted as evidence that authentic leadership produces more effective conflict management. Because both constructs were measured concurrently, the temporal direction of their relationship cannot be determined, and reciprocal relationships or shared individual and organizational determinants remain possible.

Among the individual styles, collaborating demonstrated the strongest association with overall authentic leadership. This relationship is theoretically consistent with several components of authentic leadership. Balanced processing involves consideration of relevant information and alternative perspectives before reaching decisions, which conceptually parallels the collaborative effort to integrate the concerns of different parties. Relational transparency may facilitate open discussion of disagreements, while self‐awareness may help leaders recognize how their own assumptions, emotions, and behaviors contribute to conflict. An internalized moral perspective may further provide an ethical basis for considering the legitimate interests of those involved rather than relying solely on positional authority. These conceptual parallels may help explain why collaborating showed the strongest association with authentic leadership. The finding is also consistent with previous research reporting a positive relationship between authentic leadership and collaborating conflict management [20]. Nevertheless, the observed relationship represents theoretical compatibility and statistical association rather than evidence that authentic leadership causes nurse leaders to adopt a collaborating style.

Accommodating was also positively associated with authentic leadership, and this association remained statistically significant after covariate adjustment. Theoretically, this relationship may be understood through the relational and ethical components of authentic leadership. Relational transparency emphasizes genuine interpersonal relationships, while an internalized moral perspective emphasizes behavior guided by internal values rather than solely by self‐interest. In some conflict situations, giving greater consideration to another party’s concerns may therefore be compatible with authentic leadership, particularly when preserving an important professional relationship or addressing another person’s legitimate needs is appropriate. Self‐awareness may also help leaders distinguish between situations in which maintaining their own position is necessary and those in which concession is reasonable. This interpretation does not imply that accommodation is inherently characteristic of authentic leadership or appropriate in every conflict. Rather, the positive association suggests that the two can coexist under particular circumstances. Previous studies have similarly reported positive relationships between accommodating conflict‐management approaches and authentic leadership [20, 21].

Competing, in contrast, was not significantly associated with overall authentic leadership in either the unadjusted or covariate‐adjusted analysis. This finding is theoretically informative because authentic leadership should not necessarily be equated with an absence of assertiveness. Authentic leadership emphasizes self‐awareness, transparency, balanced consideration of information, and behavior guided by internalized ethical values. A more assertive or competing response could therefore potentially coexist with authentic leadership in circumstances requiring decisive action, such as those involving patient safety, professional standards, ethical obligations, or urgent clinical decisions. Conversely, competing behavior based primarily on pursuing one’s own interests without adequate consideration of others may be less compatible with the relational and balanced‐processing components of authentic leadership. Previous studies have reported negative relationships between competing and authentic leadership [20, 22], suggesting that the relationship may vary according to context and the circumstances in which the style is used.

At the dimension level, competing showed a small positive association with balanced processing that remained statistically significant after FDR correction (*B* = 0.186, 95% CI [0.025, 0.346], *β* = 0.230, *R* ^2^ = 0.053, *p* = 0.024, *q* = 0.028). This exploratory finding should be interpreted cautiously. Balanced processing requires leaders to consider relevant information and differing perspectives before reaching a decision, but it does not necessarily require the final decision to satisfy all parties. A leader may consider competing perspectives carefully and nevertheless adopt a firm position when circumstances warrant it. However, the small proportion of explained variance and the exploratory nature of this analysis do not support a strong theoretical conclusion regarding a specific relationship between competing and balanced processing. Further research is needed to determine whether particular clinical or organizational circumstances explain this association.

Avoiding was positively associated with authentic leadership, and this association remained statistically significant after adjustment for demographic and professional characteristics. Although this finding may initially appear less consistent with authentic leadership than the associations with collaborating or compromising, the four‐component model provides a more nuanced theoretical interpretation. Self‐awareness may enable leaders to recognize when their own emotional responses could intensify a conflict, while balanced processing may sometimes require delaying an immediate response until sufficient information or alternative perspectives are available. An internalized moral perspective may also support restraint when immediate confrontation could unnecessarily damage professional relationships or interfere with a more appropriate resolution process. From this perspective, temporary avoidance may coexist with authentic leadership when it represents deliberate postponement or strategic restraint rather than disengagement from leadership responsibility.

Avoidance, therefore, should not be considered uniformly maladaptive. Nurse leaders may temporarily defer confrontation when emotions are heightened, when additional information is required, when immediate discussion could escalate the situation, or when another time or setting is more appropriate for resolution. This interpretation may also be relevant to the interpersonal and organizational context in which Saudi nurse leaders practice, where sensitivity to professional relationships, hierarchy, and workplace harmony may influence how and when conflict is addressed. However, the present study did not assess participants’ reasons for using avoidance, the specific situations in which it occurred, or the consequences of its use. A cultural or contextual explanation therefore cannot be established from these data. Although González‐García et al. [23] identified avoidance as one of the less frequently used conflict‐management approaches among nurse managers, the frequency of use does not establish whether avoidance is adaptive or maladaptive in a particular situation. The present findings consequently indicate that authentic leadership characteristics can coexist with an avoiding style rather than demonstrating that avoidance is inherently beneficial or detrimental.

Finally, compromising was positively associated with authentic leadership and remained significant after covariate adjustment. This relationship can be interpreted particularly through balanced processing, relational transparency, and self‐awareness. Balanced processing involves consideration of competing information and perspectives, which may facilitate recognition that neither party’s preferred position can always be fully maintained. Relational transparency may support open communication about disagreements and acceptable concessions, while self‐awareness may help leaders recognize the limitations of their own position. An internalized moral perspective may further provide an ethical reference point for determining which interests can reasonably be negotiated and which principles should not be compromised. Thus, compromising may be compatible with authentic leadership when it represents a transparent and considered effort to reach a mutually acceptable resolution rather than compromise for its own sake. Previous nursing research has also identified compromising as an important conflict‐management approach within healthcare work environments [24].

Taken together, the findings suggest that the relationship between authentic leadership and conflict management may be better understood as context‐sensitive rather than style‐specific. The four components of authentic leadership describe intrapersonal, relational, ethical, and information‐processing characteristics of leadership; they do not prescribe one conflict‐management response for every situation. Self‐awareness may shape the recognition of one’s own position and reactions, relational transparency may shape how disagreement is communicated, internalized moral perspective may provide an ethical basis for evaluating competing interests, and balanced processing may support consideration of different perspectives before action is taken. Different conflict situations may therefore coexist with different behavioral responses while remaining compatible with these underlying leadership characteristics. This theoretical perspective helps explain why authentic leadership was associated with collaborating, accommodating, avoiding, and compromising, whereas no significant overall association was observed with competing. Importantly, because leadership and conflict‐management characteristics were self‐reported and assessed at a single time point, these findings represent patterns of association rather than evidence of directionality, causation, or the effectiveness of any particular conflict‐management strategy. Longitudinal and context‐sensitive research is needed to examine how these relationships develop over time and whether the appropriateness and outcomes of particular conflict‐management approaches vary according to clinical urgency, hierarchical relationships, conflict intensity, and organizational context.

### 5.1. Study Implications

The observed associations suggest that authentic leadership and conflict‐management approaches are relevant, interconnected aspects of nursing leadership practice. Nursing organizations may consider addressing both constructs within leadership‐development initiatives by encouraging nurse leaders to reflect on self‐awareness, relational transparency, ethical decision‐making, balanced consideration of different perspectives, and the situational use of conflict‐management approaches. However, the present findings do not demonstrate that developing authentic leadership will cause changes in conflict‐management behavior or improve organizational outcomes. Similarly, the results do not identify a single “best” conflict‐management style because the appropriateness of a particular approach may depend on the nature, urgency, and context of the conflict. Leadership‐development programs may therefore benefit from emphasizing reflective and context‐sensitive conflict management rather than prescribing one universally preferred style. Longitudinal and intervention research is needed to determine whether changes in authentic leadership are followed by changes in conflict‐management practices and whether such changes are associated with improved staff, patient, or organizational outcomes.

### 5.2. Strengths and Limitations

This study addresses a relatively underexplored area in nursing management by examining the relationship between authentic leadership and conflict‐management styles among nurse leaders. The inclusion of nurse leaders from different geographical regions of Saudi Arabia broadened the geographical coverage of the sample. In addition, established instruments were used and demonstrated good to excellent internal consistency in the current sample.

Several limitations should be considered when interpreting the findings. First, the inclusion of nurse leaders working only in public healthcare institutions may limit the generalizability of the findings to private healthcare settings. Second, the cross‐sectional design precludes determination of temporal sequence or causality between authentic leadership and conflict‐management styles. Because both constructs were assessed concurrently, the direction of the observed relationships cannot be established, and reciprocal relationships or shared underlying determinants cannot be excluded. Third, online nonprobability convenience sampling may have introduced selection bias and limited sample representativeness. Because the number of individuals who received or viewed the invitation was unknown, a response rate could not be calculated, and the extent of potential nonresponse bias could not be assessed. Fourth, authentic leadership and conflict‐management styles were assessed concurrently using self‐report questionnaires, introducing potential common‐method and social desirability biases. Although anonymity and confidentiality were maintained, no formal assessment of common‐method bias was conducted. Shared measurement effects may therefore have influenced the observed associations. Fifth, because recruitment was conducted through online professional networks and eligibility was based on self‐report, participants’ nurse‐leader status and institutional affiliation could not be independently verified, introducing the possibility of eligibility misclassification. Sixth, the relatively small sample size (*N* = 96) may have limited statistical precision and the ability to detect smaller associations. Seventh, participants were unevenly distributed across geographical regions, with a greater proportion recruited from the Western region, which may further limit the national representativeness of the findings. Finally, the English‐only administration of both questionnaires in a multinational sample, including 44 non‐Saudi participants, may have introduced variation in item comprehension and interpretation. Although internal consistency was good to excellent, construct validity and measurement equivalence across cultural or language groups were not assessed, which warrants caution when interpreting the scores and their associations.

## 6. Conclusion

This study identified significant positive associations between self‐reported authentic leadership and collaborating, accommodating, avoiding, and compromising conflict‐management styles among nurse leaders in Saudi Arabia, whereas competing was not significantly associated with authentic leadership. The principal pattern of associations remained after adjustment for selected demographic and professional characteristics. These findings suggest that authentic leadership characteristics coexist with multiple approaches to conflict management and reinforce the importance of considering conflict‐management behavior as context‐dependent rather than assuming that one style is universally preferable. Given the cross‐sectional and self‐reported nature of the data, the findings do not establish temporal direction, causality, or the effectiveness of authentic leadership or particular conflict‐management styles. Longitudinal and intervention studies are warranted to examine how these relationships evolve and whether they are associated with subsequent workforce, organizational, or patient‐related outcomes.

## Funding

This work was funded by the Deanship of Graduate Studies and Scientific Research, Taif University.

## Conflicts of Interest

The author declares no conflicts of interest.

## Supporting Information

Additional supporting information can be found online in the Supporting Information section.

## Supporting information


**Supporting Information** STROBE Checklist.

## Data Availability

The data in this study are available from the corresponding author upon reasonable request.
