# Supplementary material for: Authentic Leadership and Conflict‐Management Styles Among Nurse Leaders in Saudi Arabia: A Cross‐Sectional Study
Source: J Nurs Manag. 2026 Nov 3;2026:7569784. doi: 10.1155/jonm/7569784 (PMC13728913; doi:10.1155/jonm/7569784)
Supplement: Supplementary file 1 [file JONM-2026-7569784-s001.docx]

**STROBE Checklist**

**Manuscript**: Authentic Leadership and Conflict Management Styles Among Nurse Leaders in Saudi Arabia: A Cross-Sectional Study

| **Section** | **Item** | **Reporting** | **Page(s)** |
| --- | --- | --- | --- |
| Title and abstract | 1a | Identify design | 1–2 |
| Title and abstract | 1b | Balanced abstract | 2–3 |
| Introduction | 2 | Scientific rationale | 4–5 |
| Introduction | 3 | Objectives and hypotheses | 5–6 |
| Methods | 4 | Design elements | 6 |
| Methods | 5 | Setting and dates | 6–7, 9–10 |
| Methods | 6a | Eligibility and selection | 6–7, 9–10 |
| Methods | 7 | Variable definitions | 8–9, 11–12 |
| Methods | 8 | Measurement procedures | 7–9 |
| Methods | 9 | Bias mitigation | 6–7, 10, 13; 29–30 |
| Methods | 10 | Sample-size rationale | 6–7 |
| Methods | 11 | Quantitative-variable handling | 8–9, 10–12 |
| Methods | 12a | Statistical methods and adjustment | 10–12 |
| Methods | 12b | Subgroups and interactions | Not applicable |
| Methods | 12c | Missing-data methods | 10 |
| Methods | 12d | Sampling-related analysis | 6, 10–12 |
| Methods | 12e | Sensitivity-analysis methods | Not conducted |
| Results | 13a | Participant counts | 10, 13 |
| Results | 13b | Reasons for nonparticipation | 10 |
| Results | 13c | Participant flow diagram | Not applicable |
| Results | 14a | Participant characteristics | 13–14 |
| Results | 14b | Missingness by variable | 10; 13–15 |
| Results | 15 | Outcome summaries | 15 |
| Results | 16a | Estimates, precision, and adjustment | 12, 15–20 |
| Results | 16b | Category boundaries | 12–14 |
| Results | 16c | Absolute-risk translation | Not applicable |
| Results | 17 | Additional analyses | 16–20 |
| Discussion | 18 | Main findings | 21 |
| Discussion | 19 | Bias and imprecision limitations | 29–30 |
| Discussion | 20 | Cautious interpretation | 21–31 |
| Discussion | 21 | External validity | 6, 29–30 |
| Other information | 22 | Funding | 37 |
